# Pannexin2 oligomers localize in the membranes of endosomal vesicles in mammalian cells while Pannexin1 channels traffic to the plasma membrane

**DOI:** 10.3389/fncel.2014.00468

**Published:** 2015-02-02

**Authors:** Daniela Boassa, Phuong Nguyen, Junru Hu, Mark H. Ellisman, Gina E. Sosinsky

**Affiliations:** ^1^National Center for Microscopy and Imaging Research, Center for Research in Biological Systems, University of CaliforniaSan Diego, La Jolla, CA, USA; ^2^Department of Neurosciences, University of CaliforniaSan Diego, La Jolla, CA, USA

**Keywords:** pannexin channels, ATP signaling, tetracysteine tag, miniSOG, correlated light and electron microscopy, electron tomography, intercellular communication, connexin

## Abstract

Pannexin2 (Panx2) is the largest of three members of the pannexin proteins. Pannexins are topologically related to connexins and innexins, but serve different functional roles than forming gap junctions. We previously showed that pannexins form oligomeric channels but unlike connexins and innexins, they form only single membrane channels. High levels of Panx2 mRNA and protein in the Central Nervous System (CNS) have been documented. Whereas Pannexin1 (Panx1) is fairly ubiquitous and Pannexin3 (Panx3) is found in skin and connective tissue, both are fully glycosylated, traffic to the plasma membrane and have functions correlated with extracellular ATP release. Here, we describe trafficking and subcellular localizations of exogenous Panx2 and Panx1 protein expression in MDCK, HeLa, and HEK 293T cells as well as endogenous Panx1 and Panx2 patterns in the CNS. Panx2 was found in intracellular localizations, was partially N-glycosylated, and localizations were non-overlapping with Panx1. Confocal images of hippocampal sections immunolabeled for the astrocytic protein GFAP, Panx1 and Panx2 demonstrated that the two isoforms, Panx1 and Panx2, localized at different subcellular compartments in both astrocytes and neurons. Using recombinant fusions of Panx2 with appended genetic tags developed for correlated light and electron microscopy and then expressed in different cell lines, we determined that Panx2 is localized in the membrane of intracellular vesicles and not in the endoplasmic reticulum as initially indicated by calnexin colocalization experiments. Dual immunofluorescence imaging with protein markers for specific vesicle compartments showed that Panx2 vesicles are early endosomal in origin. In electron tomographic volumes, cross-sections of these vesicles displayed fine structural details and close proximity to actin filaments. Thus, pannexins expressed at different subcellular compartments likely exert distinct functional roles, particularly in the nervous system.

## Introduction

**Figure 1:**
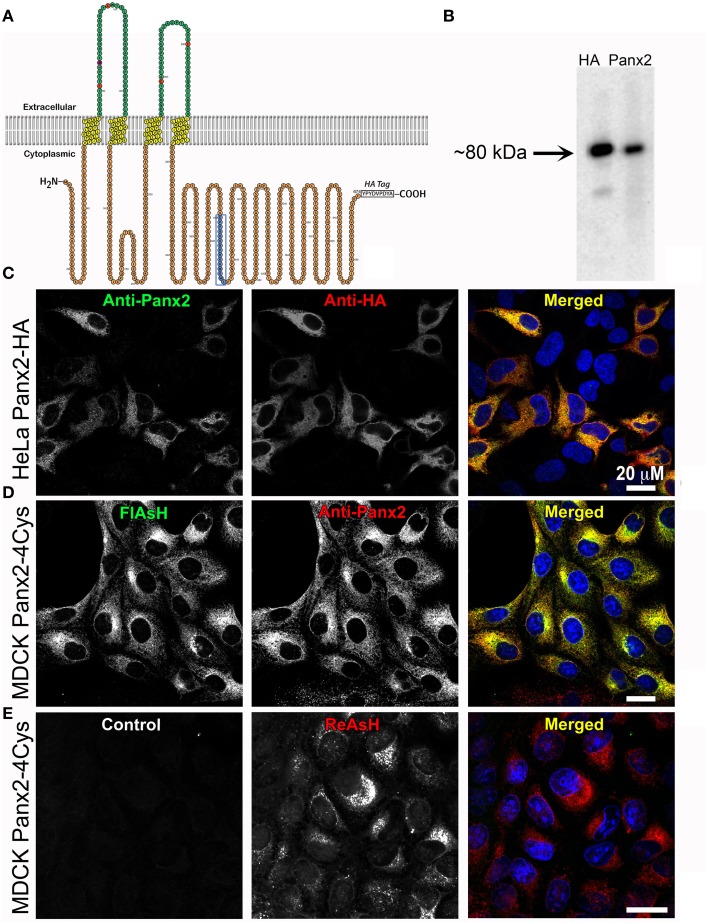
**Exogenous Panx2 is localized intracellularly in stable expressing cell lines. (A)** Schematic of the membrane topology of a Panx2 monomer. The Panx2 peptide sequence used to generate the carboxy terminal antibody used in this study is indicated in blue as well as an appended HA tag. **(B)** Western blot of a cell lysate from HeLa cells stably expressing Panx2-HA. Both the anti-Panx2 (polyclonal) and anti-HA (monoclonal) antibodies detect a ~80-kDa band corresponding to the molecular mass of Panx2-HA. **(C–E)** Images are single plane confocal micrographs. **(C)** In this HeLa cell line, immunofluorescence demonstrates almost complete overlap of anti-Panx2 (left image) and anti-HA antibodies (middle image). Anti-Panx2 is displayed in green, anti-HA in red and DAPI is blue in the merged images for **(C)** (right column). **(D)** MDCK cells stably expressing Panx2-4Cys stained with FlAsH (green) and the anti-Panx2 antibody (red) also show almost complete overlap of the two fluorescent signals. **(E)** In this panel, no primary antibody was used in the initial incubations (control, left image), while ReAsH staining (red) confirmed the presence of Panx2-4Cys (middle image).

The pannexins (Panx1, Panx2, and Panx3) are unique entities in the “connexin-like” family of proteins. These three membrane proteins are topologically similar to innexins (invertebrate gap junction proteins) (Panchin et al., 2000; Locovei et al., 2006a) with some sequence similarities, but function as single membrane channels (pannexons) in mammals (Locovei et al., 2006a). As shown in Figure 1A, the hallmarks of this folding topology are the four transmembrane segments, conserved cysteine residues found in the two extracellular loops, and the cytoplasmic domains comprised of the amino and carboxy termini and the connecting loop between transmembrane segments two and three. Based on these similarities to connexins, the transmembrane segments most likely are α-helical. However, unlike connexins, pannexins contain four strictly conserved cysteines instead of six (Ambrosi et al., 2010) and an N-linked glycosylation site in one of the two extracellular loops (N254 for Panx1, putative site N86 for Panx2, and N71 for Panx3) (Boassa et al., 2007; Penuela et al., 2013). Pannexins oligomerize to form channel structures also called pannexons.

Functionally, Panx1 and Panx3 have both been shown to perform a role in paracrine intercellular signaling. The ubiquitous Panx1 forms an ATP release channel that can interact with specific P2 purinergic receptors as part of an ATP signaling pathway (Locovei et al., 2006b; Pelegrin and Surprenant, 2006), but also can be differentially modulated by voltage and K^+^ (Wang et al., 2014). Panx1 is mechanosensitive, a factor important in calcium wave propagation (Locovei et al., 2006b). Several studies have established that Panx1 channels are part of adaptive or inflammasome responses in the immune system (Kanneganti et al., 2007; Silverman et al., 2008; Schroder et al., 2010; Maslieieva and Thompson, 2014). Panx1 channels play an important role in the nervous system since they are expressed in significant levels in neurons and astrocytes (Vogt et al., 2005; Weickert et al., 2005; Ray et al., 2006; Huang et al., 2007; Bargiotas et al., 2011; Cone et al., 2013). During ischemia and subsequent anoxic depolarization, Panx1 channels in neurons are activated by NMDA receptors and open releasing ATP and glutamate (Thompson et al., 2008). This activation of neuronal Panx1 channels can occur in response to nitric oxide or Src activation of NMDA receptors, as recently reviewed by Thompson (2014).

Panx3, which is more similar to Panx1 than to Panx2 in terms of molecular mass and its channel characteristics, localizes to the plasma membrane as the fully glycosylated membrane channel (Penuela et al., 2009); Panx3 is thought to be expressed mostly embryonically or only in skin or cartilage (Panchin et al., 2000; Bruzzone et al., 2003; Baranova et al., 2004), although published immunoblot analyses showed weak bands with a second higher molecular weight species present in a broad range of tissues (Penuela et al., 2007; Langlois et al., 2014). It has been proposed that Panx3 channels in cartilage switch the chondrocyte cell fate from proliferation to differentiation by regulating the intracellular ATP/cAMP levels (Iwamoto et al., 2010).

Because combinations of connexin isoforms can form heteromeric hemichannels or heterotypic intercellular channels, early research investigated whether the same was true for pannexins after they were first identified. Bruzzone et al. (2003) originally reported that co-injection of Panx1 and Panx2 mRNA in unpaired and paired Xenopus oocytes resulted in functional hemichannels and intercellular channels, respectively with properties distinct from Panx1 homomeric hemichannels and intercellular channels. However, a subsequent papers by Naus' group (Lai et al., 2009; Le Vasseur et al., 2014) reported *only* intracellular localizations of Panx2 in mammalian cells. A different study concluded that Panx2 is found at the plasma membrane when co-expressed with Panx1 in NRK or HEK 293T cells (Penuela et al., 2009), however, within light microscopic resolution, it is unclear if in these overlapping areas of staining, Panx1 and Panx2 make heteromeric channels or form mixed populations of homomeric channels. We previously showed that preparations of Panx1 and Panx2 purified from baculovirus infected Sf9 cells made stable homomeric functional channels but unstable heteromeric channels (Ambrosi et al., 2010). Presumably an oligomerization mis-match occurs because Panx1 formed a homomeric hexamer while homomeric Panx2 pannexons were octameric (Ambrosi et al., 2010). We also found that the individual characteristics of Panx1 and Panx2 homomeric channel openings when expressed in Xenopus oocytes were different from each other, again suggesting that these two isoforms create two different kinds of pannexons (Ambrosi et al., 2010). A recent study demonstrated that when Panx1 and Panx2 channels expressed in Xenopus oocytes were stimulated to a putative open state, there was significantly less membrane currents or Yo-Pro dye uptake of Panx2 channels as compared to Panx1 channels (Hansen et al., 2014). The authors reasoned that either Panx2 channels required different physiological conditions from Panx1 to open or Panx2 is expressed at low levels at the plasma membrane.

As described in this study, using differential labeling and imaging approaches in immortalized tissue culture cells we observed that Panx1 and Panx2 channels had different sub-cellular localizations. Subsequently, we addressed this question using light microscopic imaging of endogenous pannexins in native brain tissue complemented by correlated light and electron microscopic studies (CLEM) using EM compatible genetically encoded probes that allow investigation of the distribution of Panx2 at significantly higher resolution than conventional fluorescence microscopy. We report here that Panx1 and Panx2 were differentially localized both in neurons and astrocytes in the adult mouse brain. Recombinant protein expression in different cell lines confirmed these observations of segregated Panx1 and Panx2 sub-cellular localizations. Previously, our group and others showed that Panx1 is fully N-glycosylated and transported to the cell membrane (Boassa et al., 2007, 2008; Penuela et al., 2007). In contrast, we present data here that Panx2 has an intracellular localization in the membrane of cytoplasmic endosomal vesicles and exists as a partially-glycosylated species. The resolution provided by electron microscopy suggests that Panx2 pannexons could operate as vesicular channels that are in transport to the cell membrane.

## Materials and methods

### Antibodies and reagents

Below we provide antibody identification numbers in The Antibody Registry, http://antibodyregistry.org/ for the antibodies used in this study.

#### Pannexin antibodies

Panx1 and Panx2 antibodies were generated against peptides using sequences in the N-terminus (Panx1 mouse monoclonal; N-terminus (LKEPTEPKFKGLRLE characterization fully described in Cone et al. (2013) and the C-terminus (Panx2 rabbit polyclonal; (EPPVVKRPRKKMKWI, amino acids 420–434 Figure 1A). These peptides and anti-peptide antibodies were custom produced and purified by Abgent, Inc. (San Diego, CA). These antibodies recognize invariant sequences in rodent and human pannexins. The chicken anti-Panx1 antibody with a distal C-terminal epitope was provided by Dr. Gerhard Dahl (Locovei et al., 2006a) and our characterizations of it are documented in Cone et al. (2013). Panx2 antibodies were used at dilutions of 1:15,000 for western blots and 1:250–1:500 for immunofluorescence experiments.

#### Anti-HA antibodies

In this study, we used a monoclonal HA tag antibody (Sigma Aldrich, St. Louis, MO Catalog Number H9658, Antibody Registry ID AB_260092) for both immunofluorescence and western blots. Dilutions used were 1:10,000 for western blots and 1:250–1:500 for immunofluorescence experiments.

#### Cellular/organellar markers

The following antibodies were used as markers of subcellular compartments: anti-Rab4 (BD Biosciences Catalog Number 610888, Antibody registry ID AB_398205), anti-Clathrin (BD Biosciences Catalog Number 610499, Antibody registry ID AB_397865), anti-p47a/AP3M1 (BD Biosciences Catalog #610900, AB_10015260) and anti-adaptin β (BD Biosciences Catalog #610381, Antibody Registry ID AB_397765). We used anti-GFAP antibodies (Advanced Immunochemical Incorporated Catalog #031223—Lot 1gf, Antibody registry ID AB_2314538). Antibodies were used at 1:250–500 dilutions.

### DNA constructs

All constructs in this paper use amino acid sequences for rat pannexins (Uniprot accession codes P60571 and E0X643 for Panx2 and Panx1, respectively). The schematic of the Panx2 sequence shown in Figure 1A was made using the web-based program Protter (Omasits et al., 2014) http://wlab.ethz.ch/protter/start/. The rat Panx2 construct used in this study is described more in detail in Ambrosi et al. (2010). Methods for development and characterization of tagged Panx1 and Panx2 stably expressing mammalian cell lines are described in Boassa et al. (2007) and Boassa et al. (2010).

### Cell lines, transfection, and transduction methods

MDCK, HeLa, and HEK 293T cells were cultured in Dulbecco's modified Eagle's medium (Mediatech, Inc., Manassas, VA) supplemented with 10% FBS in a 37°C incubator with 10% CO_2_. Transient transfections were carried out using Lipofectamine 2000 reagent (Life Technologies, Carlsbad, CA) following the manufacturer's protocol and 0.5 micrograms of tagged/untagged Panx2 DNA subcloned into a pcDNA3.1 vector. The transfection mixture was added to the cells plated onto 6-well dishes containing poly-d-lysine-coated coverslips or onto poly-d-lysine-coated glass bottom culture dishes (MatTek, Ashland, MA) for CLEM. Cells were incubated for 4–6 h after which the transfection medium was removed and replaced with regular growth medium. Transfected cells grew for 24–48 h post-transfection, then were fixed for either light microscopy or CLEM (both fixation conditions described below) and prepared for fluorescent imaging.

Transductions were carried out using a retroviral system according to the protocols from the Nolan laboratory (www.stanford.edu/group/nolan). Untagged or tagged-Panx2 was subcloned into a modified version of the mammalian retroviral vector pCLNCX-Hygro (originally obtained from Imgenex, La Jolla, CA). Experiments were conducted on stably untagged and tagged Panx2-expressing cell lines generated by transduction followed by selection with the antibiotic hygromycin (Gibco-BRL, Life Technologies).

### Western blots for tissue culture cells

MDCK, HeLa, and HEK 293T cells stably expressing untagged or tagged Panx2 proteins grown on Petri dishes were washed three times with Hanks' balanced salt solution pre-warmed at 37°C. Proteins were extracted from cells in SDS buffer containing 4% beta-mercaptoethanol, 1 mM phenylmethylsulfonyl fluoride, and a protease inhibitor mixture (Sigma Aldrich). Whole cell lysates were separated by SDS-PAGE on a 4–20% Tris-Glycine gel (Life Technologies) and then electrophoretically transferred to a PVDF membrane (Millipore, Bedford, MA). Membranes were blocked overnight in 5% non-fat dry milk made in 0.5% Tween-20 in PBS (PBS-T) and incubated with primary antibodies for 1 h at room temperature. Membranes were then washed in PBS-T to remove excess primary antibody, incubated in horseradish peroxidase-linked secondary antibodies in 5% non-fat dry milk in PBS-T for 1 h at room temperature and finally rinsed 3 times for 10 min each in PBS-T. To visualize proteins, the membrane was processed for enzyme-linked chemiluminescence using an ECL Kit (Amersham Biosciences).

### Analysis of post-translational modifications of Panx1 and Panx2 expressing tissue culture cells

Whole cell lysates from HEK 293T, HeLa and MDCK cells stably expressing Panx2-HA, Panx2-WT, or Panx1-WT proteins were incubated with 10 units of calf intestinal alkaline phosphatase (CIAP) for 5 h at 37°C; or with 1500 units of *N*-glycosidase F (PNGase F) (New England Biolabs, Beverly, MA) for 5 h at 37°C at pH 7.5 following procedures we used in Boassa et al. (2007) or 50 μM pan-caspase inhibitor Z-VAD-OMe-FMK for 5 h at 37°C (Millipore, Billerica, MA) following a procedures described in Penuela et al. (2014). Samples were boiled for 5 min, and western blotted as described above.

### Immunofluorescence and confocal imaging

Tissue culture cells plated on poly-d-lysine coated coverslips were fixed in 4% paraformaldehyde in phosphate-buffered saline (PBS) for 15 min, washed in PBS, permeabilized in 0.1% Triton X-100, and blocked in 1% BSA, 50 mM glycine and 2% normal serum. The primary antibodies were mixed in blocking buffer diluted five-fold in PBS, and applied for 1 h at room temperature. The secondary antibodies were diluted in the same buffer, and applied for 1 h at room temperature. Confocal immunofluorescence images (1024 × 1024 pixels) were acquired on the Olympus Fluoview 1000 laser scanning confocal microscope using a 60X oil immersion objective with numerical aperture 1.42.

### Method for colocalization analysis

For each set of images where we were interested in quantifying overlaps of fluorescence signals, a Manders' coefficient for colocalization was calculated using the JACoP plugin (Bolte and Cordelieres, 2006), a colocalization analysis tool for ImageJ (rsb.info.nih.gov/ij). Here, we used the Manders' coefficient as a measure of how much the pixel intensities in one channel match with pixel intensities in the same location in the other channel. The Manders' coefficient quantifies the degree of overlapping of two fluorophores and varies for 0–1, where 0 corresponds to non-overlapping and 1 reflects 100% colocalization. The average Manders' coefficient and its standard deviation were analyzed for multiple images of each sample, taking the number of cells in each image into consideration (i.e., Manders' coefficient for each image is weighted by the number of cells).

### Statistical analysis

We used the Prism software package (GraphPad, La Jolla CA) to test for statistical significance levels between samples. In particular, an unpaired *t*-test for pair-wise comparisons with Welch's correction for unequal standard deviations and with a significance cutoff set to α = 0.05 was applied to the Manders' coefficients of our co-localization studies.

### Preparation of rodent brain tissue for immunohistochemistry and western blots

All experiments involving vertebrate animals conform to the National Institutes of Health *Guide for the Care and Use of Laboratory Animals* and were approved by the Institutional Animal Care and Use Committee of the University of California San Diego. The animal welfare assurance number is A3033-01.

#### Immunohistochemistry

Animals were fully anesthetized with an intraperitoneal injection of pentobarbital, and oxygenated Ringer's solution at 37°C containing xylocaine and heparin was perfused transcardially for 3 min followed by 4% paraformaldehyde for 10 min. The brain was removed and post-fixed in 4% paraformaldehyde overnight at 4°C. Sagittal sections were cut on a Leica vibratome at a thickness of 50 microns and stored at −20°C in cryoprotectant solution (30% glycerol, 30% ethylene glycol in PBS) until processed for immunohistochemistry. Free-floating tissue sections were blocked with 4% normal donkey serum, 1% bovine serum albumin, 1% cold water fish gelatin, 0.5% Triton X-100, and 50 mM glycine in PBS for 1 h at room temperature. Primary antibodies were mixed in blocking buffer diluted five-fold in PBS, and then applied overnight at 4°C. FITC, Rhodamine RedX, and Cy5 conjugated donkey secondary antibodies (Jackson ImmunoResearch Laboratories, Inc., West Grove, PA) were applied for 2.5 h at room temperature. The immunolabeled tissue samples were carefully mounted as flat as possible using gelvatol as mounting medium. Fluorescence imaging was performed on an Olympus Fluoview 1000 laser scanning confocal microscope using a 40X oil immersion objective with numerical aperture 1.3, or a 60X oil immersion objective with numerical aperture 1.42.

#### Western blots

Animals were fully anesthetized with an intraperitoneal injection of pentobarbital, and oxygenated Ringer's solution at 37°C containing xylocaine and heparin was perfused transcardially. The brain was dissected and brain lysates were then prepared by tissue homogenization in RIPA buffer containing 1X complete protease inhibitor cocktail (Roche). The volume of buffer was adjusted with the weight of tissue (2–3 mg per 10 μl). Tissue and cell debris was removed by centrifugation. The lysate was boiled for 5 min in 1X SDS sample buffer containing 4% beta-mercaptoethanol and 1 mM phenylmethylsulfonyl fluoride, and then loaded on a 4–20% Tris-Glycine gel (Life Technologies), and transferred to a PVDF membrane (Millipore, Bedford, MA). Membranes were blocked overnight in 5% non-fat dry milk and 0.5% Tween-20 in PBS, and incubated for 1 h with primary antibody at room temperature. After extensive washes in 0.5% Tween-20 in PBS, membranes were incubated for 1 h with secondary antibody at room temperature. Western blots were developed with an ECL kit (Amersham Biosciences).

### Tetracysteine labeling, photooxidation of diaminobenzidine (DAB) and electron microscopy (EM)

We used protein fusions with either the tetracysteine tag (FLNCCPGCCMEP) liganded with FlAsH or ReAsH for fluorescence imaging (Martin et al., 2005) or the weakly fluorescent MiniSOG (Shu et al., 2011), both developed by Roger Y. Tsien's laboratory for *in situ* protein localization. The ReAsH-labeled Panx2-4Cys proteins, or the Panx2-MiniSOG fusion proteins were photooxidized in the presence of DAB and oxygen for protein staining for EM as described in several of our publications (Boassa et al., 2007, 2008, 2013).

### EM tomography

MDCK cells stably expressing Panx2-4Cys were stained with ReAsH-EDT_2_, fixed, photooxidized, and processed for electron microscopy (EM) as described in Boassa et al. (2013). Half micron sections were cut from Durcupan embedded specimens using a diamond knife and then coated with carbon on both sides. Colloidal gold particles (10 nm diameter) were deposited on each side to serve as fiducial markers. For reconstruction, three tilt series of images were recorded at regular tilt (angular increments of 2° from −60° to +60° increments) with a JEOL 4000EX intermediate high-voltage electron microscope operated at 400 kV. The specimens were irradiated before initiating a tilt series to limit anisotropic specimen thinning during image collection. Tilt series were recorded using a 4 × 4 k custom high resolution slow-scan CCD camera system delivering 25% contrast at Nyquist. A rough alignment for each tilt series was calculated using the IMOD package (Kremer et al., 1996). Fine alignment of projections and combination of the three tilt series data for the final 3D reconstruction were performed using the TxBR reconstruction package (Lawrence et al., 2006). Reconstructed volumes were viewed and vesicles segmented using the automated thresholding algorithm in Amira (FEI Visualization Sciences Group, Burlington, MA). Tomogram animations were generated using Amira. Single particle 2D class averages of 2D cross-sections through vesicle tomographic slices were obtained using the EMAN2 software package (Tang et al., 2007), which was also used to measure vesicle diameters. All image data, tomograms, segmentations and full-resolution movies for the tomogram presented in **Figure 7** can be accessed for downloading in the Cell Centered Database (http://www.ccdb.org) (CCDB Microscopy Product ID:48720; Project ID:2010) and Cell Image Library (www.cellimagelibrary.org).

## Results

In this study, we examined the sub-cellular distributions of Panx2 oligomers and how these localizations differ from those of Panx1 channels. In previous publications, we had established that Panx1 channels are found at the plasma membrane and in intracellular stores characteristic of anterograde protein trafficking (Boassa et al., 2007, 2008; Dolmatova et al., 2010). Published studies of sub-cellular localizations of Panx2 using fluorescence microscopy are contradictory. Some studies indicated Panx2 distribution both at the cell membrane and intracellular locations (Penuela et al., 2009; Swayne et al., 2010) and others reported that Panx2 was only in the cytoplasm (Lai et al., 2009; Wicki-Stordeur et al., 2013; Le Vasseur et al., 2014). Thus, as part of this study we extended experiments to electron microscopic imaging in order to gain resolution and cellular context.

### Anti-Panx2 antibody localizations of exogenously expressed tagged Panx2 and antibody validation

As a first step, we developed a polyclonal antibody against a carboxy terminus peptide composed of amino acids 420–434 (see blue amino acids and box in the Panx2 topology model in Figure 1A) and cell lines stably expressing Panx2 with either an HA tag or a tetracysteine domain (4Cys) appended to the C-terminus. These genetic epitope tags served both as aids in localization and as validation tests for our anti-Panx2 antibody. As demonstrated in Figure 1B, staining of western blots of the same cell lysates from a HeLa cell line stably expressing Panx2-HA predominantly contained a band at about 80 kDa identified by both a monoclonal anti-HA antibody and our polyclonal anti-Panx2 antibody. It is worth noting that Penuela et al. (2014) have reported that Panx2 exhibited caspase cleavage with caspase 3 or caspase 7 treatment resulting in a band of ~36 kDa. For our constructs, the Panx2 caspase cleavage site is located N-terminally to our antibody epitope and the HA tag, so it is possible that bands of a size ~36-38 kDa in molecular mass could represent a caspase cleaved Panx2. Bands greater than 80 kDa should indicate Panx2 post-translational modifications.

Using this Panx2 antibody and epitope tagged Panx2, subcellular localizations were investigated by confocal microscopy with Panx2-expressing HeLa or MDCK cells. Confocal immunofluorescence images of Panx2-HA -expressing HeLa cells (Figure 1C) showed almost complete overlap of FITC (Anti-Panx2, Figure 1C left image) and Rhodamine RedX (Anti-HA, Figure 1C, middle image) fluorescence when merged (Panx2 = green; HA = red, right image), as indicated by the yellow signal. Nuclei were stained with DAPI (blue) for better identification of cells. As an additional validation, MDCK cells stably expressing Panx2-4Cys were stained with anti-Panx2 antibodies and the FlAsH reagent that specifically binds to the 4Cys tag (Gaietta et al., 2002). In the merged image in Figure 1D (right image), the labeling from the green FlAsH (left image) and red anti-Panx2 (middle image) were coincident as displayed by the intense yellow color. Following the same antibody labeling procedure, but using a solution lacking the Panx2 primary antibody resulted in images devoid of fluorescent signal (Figure 1E, left image, control). Images of ReAsH labeling showed intracellular fluorescence indicating the presence of Panx2-4Cys (Figure 1E, middle, right images) and the specificity of our anti-Panx2 antibody.

### Panx2 protein sizes in brain tissue differs from model cell lines

**Figure 2:**
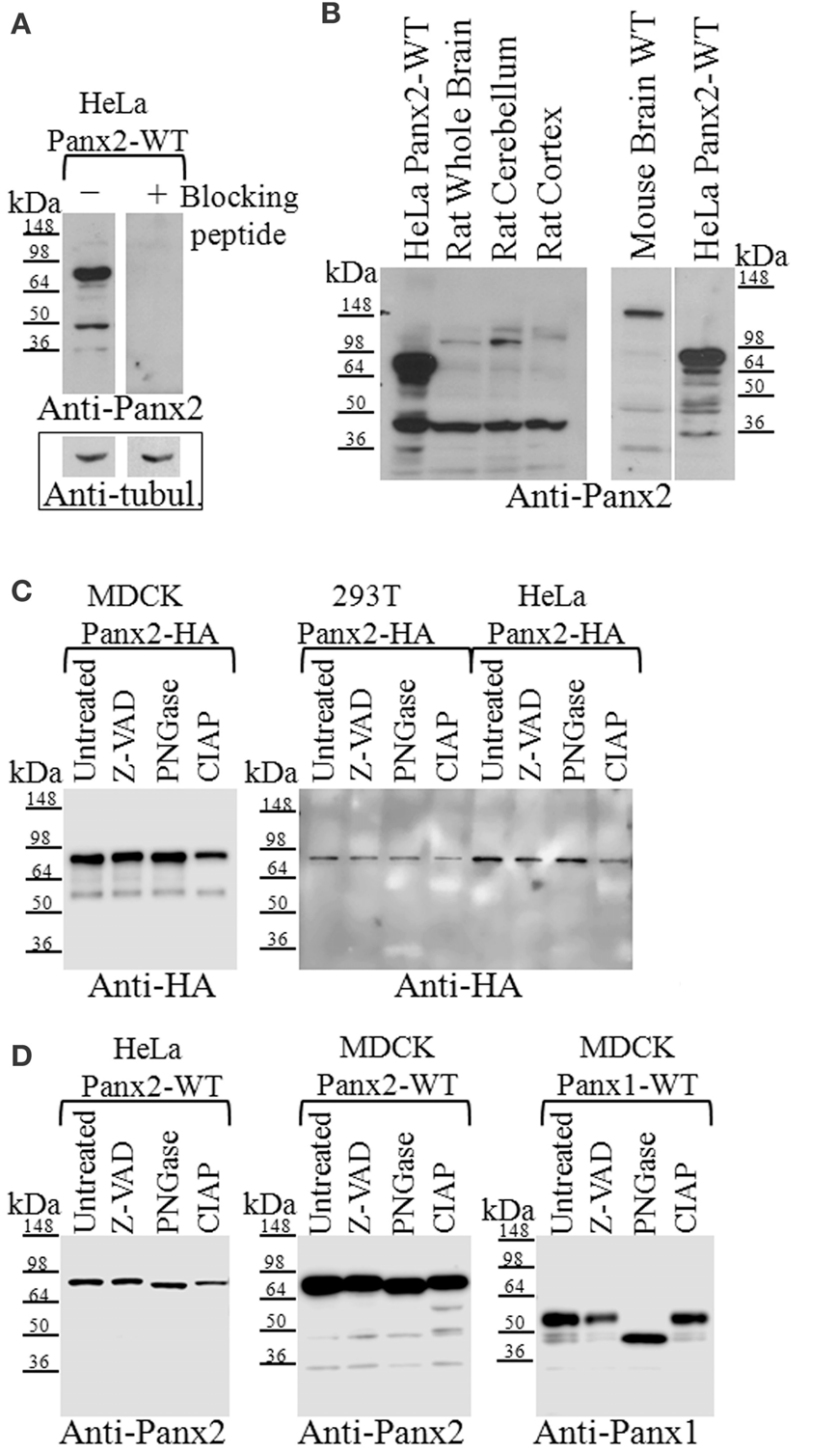
**Western blot analysis of Panx2 in tissue and post-translational modifications in tissue culture cells. (A)** Incubation of the Panx2 antibody with the immunizing peptide prior to western blotting eliminated the labeling of untagged Panx2-WT proteins stably expressed in HeLa cells. Western blots for alpha-tubulin levels shown below the Panx2 immunoblots served as loading controls for these lysates. **(B)** The expected mass of Panx2 monomers based on their amino acid sequence is ~74 kDa and western blot analysis of HeLa cell lysates stably expressing untagged rat Panx2 showed a major band at about this molecular mass (left and right hand lanes). Some lower bands were also observed at about 45 kDa. In tissue lysates from rat and mouse brains, a weaker ~74 kDa band was observed as well as few faster migrating bands that may represent caspase cleaved Panx2 species. **(C)** Cell lysates from three cell lines, MDCK, HEK 293T, and HeLa, each stably expressing HA-tagged Panx2, were treated with either Z-VAD-OMe-FMK (Z-VAD) to inhibit caspase-dependent cleavage, PNGase to reduce glycosylation, or CIAP for protein dephosphorylation. We did not see any significant shift in the bands indicating that at least in these cell types, HA-tagged Panx2 is not highly phosphorylated or glycosylated. **(D)** However, cell lysates from HeLa, and MDCK, each stably expressing untagged Panx2-WT revealed a slight shift in banding pattern following PNGase treatment. As positive control, lysates from MDCK cells stably expressing untagged Panx1-WT showed significant band shifts after PNGase treatment. Western blots shown here are representative of at least three independent experiments per group.

We confirmed that our polyclonal Panx2 antibody was specific for Panx2 by incubating this antibody with the immunizing peptide, which effectively abolished western blot signal detection (Figure 2A). Comparison of western blots of rat and mouse brain lysates using our anti-Panx2 antibody revealed a weak band similar in size to the expected ~74 kDa we observed in western blots of HeLa cells stably expressing exogenous untagged Panx2 wild type proteins (Figure 2B). A faster migrating strong band at about 45 kDa was also detected as well as 2–4 higher molecular mass bands. These lower bands were not due to cross-reactivity of the Panx2 antibodies with Panx1 as immunoblotting of lysates from endogenously expressing Panx1 MDCK cells did not show any bands (data not shown). Lower bands could result from caspase cleavage occurring during tissue collection. However, to prevent caspase-dependent cleavage we treated our lysates with the pan-caspase inhibitor ZVAD (Figure 2C). No differences were detected in the banding pattern when compared to untreated samples suggesting that the presence of lower bands detectable either by Anti-HA or Anti-Panx2 antibody in some preparations could be the result of non-specific protein degradation.

### Post-translational modifications of Panx2 in tissue culture cells

While Panx1 and Panx3 have N-linked glycosylation sites that allow for trafficking to the plasma membrane (Boassa et al., 2007; Penuela et al., 2007), Panx2 has a putative glycosylation site at N86 that has been reported to exhibit partial glycosylation (Penuela et al., 2009) where the band shift is far less than a fully glycosylated form (as compared with Panx1). In Figure 2C we show immunoblot analysis of lysates from stably expressing Panx2-HA MDCK, HEK 293T and HeLa cells treated either with PNGase to determine its N-linked glycosylation status or with CIAP for dephosphorylation. We found that both treatments did not significantly shift the banding pattern, indicating that in our Panx2-HA samples glycosylation and phosphorylation were not detectable. However, lysates from Panx2-WT (untagged) proteins stably expressed in HeLa or MDCK cells revealed a small shift in the bands following PNGase treatment, similarly to what has been previously shown (Penuela et al., 2009) suggesting that the untagged Panx2 proteins exists both as a non-glycosylated core protein (GLY0) and a high mannose-type glycoprotein (GLY1) while the Panx2-HA exists only as a non-glycosylated core protein (Figure 2D). Interestingly, following CIAP treatment two new bands appeared at about 60 and 50 kDa in the lysates from Panx2-WT (untagged) proteins, although this seemed to vary among cell lines. As a positive control, lysates from MDCK cells stably expressing Panx1-WT (untagged) proteins showed a change in banding pattern following PNGase treatment (Figure 2D).

### Cellular distributions of Panx1 and Panx2 are non-overlapping in cell cultures and brain tissue

**Figure 3:**
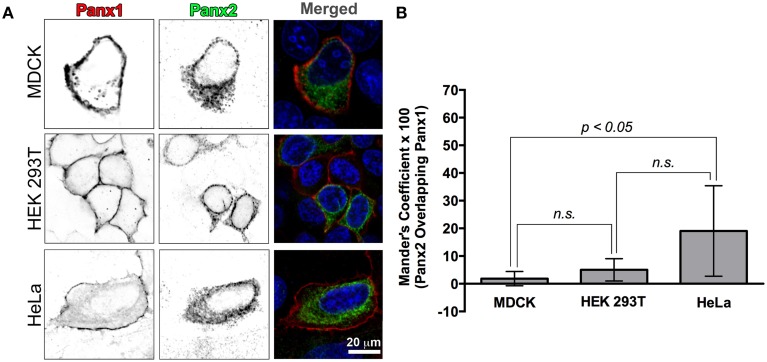
**Recombinant Panx1 and Panx2 have non-overlapping cellular distributions in various cell lines. (A)** MDCK, HEK 293T, and HeLa cells were co-transfected with wild type untagged Panx1 and Panx2. Images are single confocal slices. In the single channel confocal images (left and middle columns), the color table has been inverted for better visualization of the individual fluorescence channels and in particular, vesicle populations. Panx1 was labeled with a mouse monoclonal antibody (Cy5 secondary antibody detection, shown in red in the merged image) while our polyclonal C-terminal Panx2 antibody was used to stain Panx2 (FITC secondary detection, shown in green in the merged image). In all three cell types, Panx1 was found mostly in the plasma membrane (left column, red color in the “Merged” images in the right hand column) while Panx2 remained in intracellular locations (middle column, green color in the Merged images in the right hand column). In the right hand column images (“Merged”), the nuclei are stained with DAPI (blue). We calculated a Manders' colocalization coefficient in order to quantify the degree of overlap of Panx1 and Panx2 in these cell lines. The graph of average percent overlap (as indicated by the Manders' coefficient × 100) shown in **(B)** demonstrates that there is very little overlap (20% and below) between Panx1 and Panx2 in these three cell types with significantly higher overlap in HeLa cells. The numbers of images analyzed were 8, 7, and 11 for HEK 293T cells, HeLa cells and MDCK cells, respectively. Error bars indicate standard deviations. (n.s., not significant).

While Panx2 oligomers have been reported to traffic to the plasma membrane when co-expressed with Panx1 (Penuela et al., 2009), we found that co-expressing untagged Panx1 and Panx2 in various cell lines resulted in non-overlapping populations of Panx1 and Panx2. As shown in Figure 3A, when co-expressed in three different cell lines (MDCK, HEK 293T, and HeLa cells), Panx1 had strong plasma membrane fluorescence while Panx2 labeling was localized into intracellular compartments. For these experiments we used untagged, wild type Panx1 and Panx2, and immunolabeling was performed with a monoclonal Panx1 antibody and the polyclonal Panx2 antibody described in the previous section. We also obtained the same results in experiments with myc and HA tagged versions of Panx1 and Panx2, respectively, expressed in these cell types (data not shown) and also in our initial experiments using a chicken antibody provided by Gerhard Dahl (Locovei et al., 2006a; Cone et al., 2013).

In order to quantify the overlap between Panx1 and Panx2 fluorescence, we calculated the Manders' coefficient for these single plane confocal images (Figure 3B). Manders' coefficients for colocalization for HEK 293T and MDCK cell images (number of analyzed images were 8 and 11, respectively) were below 0.05 (5% colocalization), while a similar analysis of 7 HeLa cell confocal images had an average Manders' coefficient of about 0.2 (20% colocalization).

**Figure 4:**
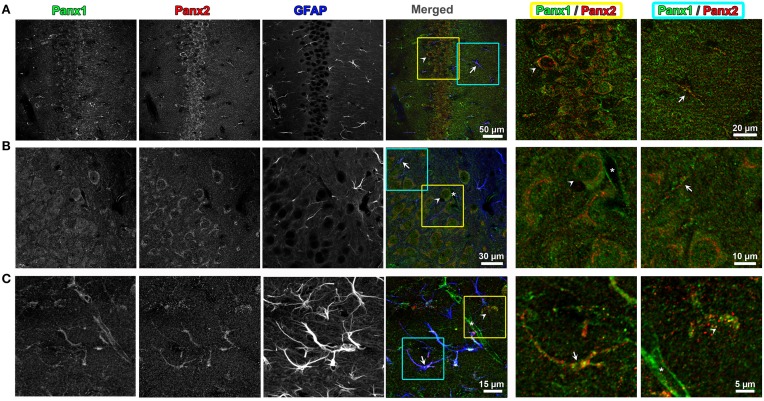
**Endogenous Panx1 and Panx2 show non-overlapping signals in hippocampal neurons and astrocytes**. Confocal images of the CA1 field of the hippocampus in the adult mouse brain are shown in **(A–C)**. Images in **(A,B)** are single confocal planes while **(C)** represents maximum intensity projections of confocal image stacks to better show the astrocyte morphology. The tissue has been immunolabeled for Panx1 (left black and white image column), Panx2 (middle black and white image column) and the astrocytic marker, GFAP (right black and white image column). In the color image column labeled “Merged” (middle column of figure), the three black and white images were superimposed with the Panx1, Panx2, and GFAP labelings displayed in green, red and blue, respectively. Three-fold enlargements of two areas highlighted by yellow and cyan boxes in each of these merged images are displayed in the two far right columns (*cyan = far right column, yellow = column left of right hand column*). These enlargements only display the Panx1 (green) and Panx2 (red) signal to better show the cellular segregation of Panx1 and Panx2 in CA1 pyramidal cells and astrocytes. Example astrocytes are indicated by arrows, neurons by arrowheads and capillaries by an asterisk. Note that in astrocytes and neurons there is little overlap in populations of Panx1 and Panx2. Also, the capillaries contain Panx1, but not Panx2.

Because previous studies based on *in situ* hybridization of Panx2 mRNA have indicated that the CNS is enriched in Panx2 (Bruzzone et al., 2003; Vogt et al., 2005; Zappala et al., 2007; Bargiotas et al., 2011), we looked for the segregation and overlap of Panx1 and Panx2 within this tissue. Triple labeling of 50 μm thick mouse brain sections with Panx1, Panx2, and glial fibrillary acidic protein (GFAP) antibody, a specific marker for astrocytes, demonstrated that the two Panx isoforms localize at different subcellular compartments in both astrocytes and neurons. In CA1 hippocampus (Figure 4), we observed that Panx2 was highly localized to pyramidal cell somata while Panx1 was distributed in cell bodies as well as in axons (*see enlargement of yellow and blue boxed area in Panel A*). In neuronal cell somas, Panx2 labeling did not overlap with Panx1 and was mainly perinuclear while the Panx1 signal was localized to intracellular populations with little plasma membrane staining (*Panel B*). In astrocytes (*as exemplified by the astrocyte in Panel 4C blue and yellow boxed area*), Panx1 and Panx2 were both found as discrete punctate spots along the astrocytic processes defined by GFAP (Bushong et al., 2002). These patterns of Panx1/Panx2 segregation were typical of other areas of the brain including cerebellum and cerebral cortex (data not shown). Furthermore, Panx1 was expressed in the capillaries and blood vessels throughout the brain, confirming previous reports (Burns et al., 2012; Gaete et al., 2014). Our mouse brain stainings typically showed more Panx1 immunofluorescence in blood vessels than similar immunofluorescence imaging of Panx1 in rat brain blood vessels (Cone et al., 2013). However, we noted in Cone et al. (2013) that the level of vessel staining was dependent on the primary antibody used for labeling. More importantly and relevant to this study was that we never found Panx2 immunofluorescence in cerebral blood vessels.

### Correlated light and EM (CLEM) imaging of photooxidized Panx2-miniSOG reveals Panx2 in intracellular compartments

**Figure 5:**
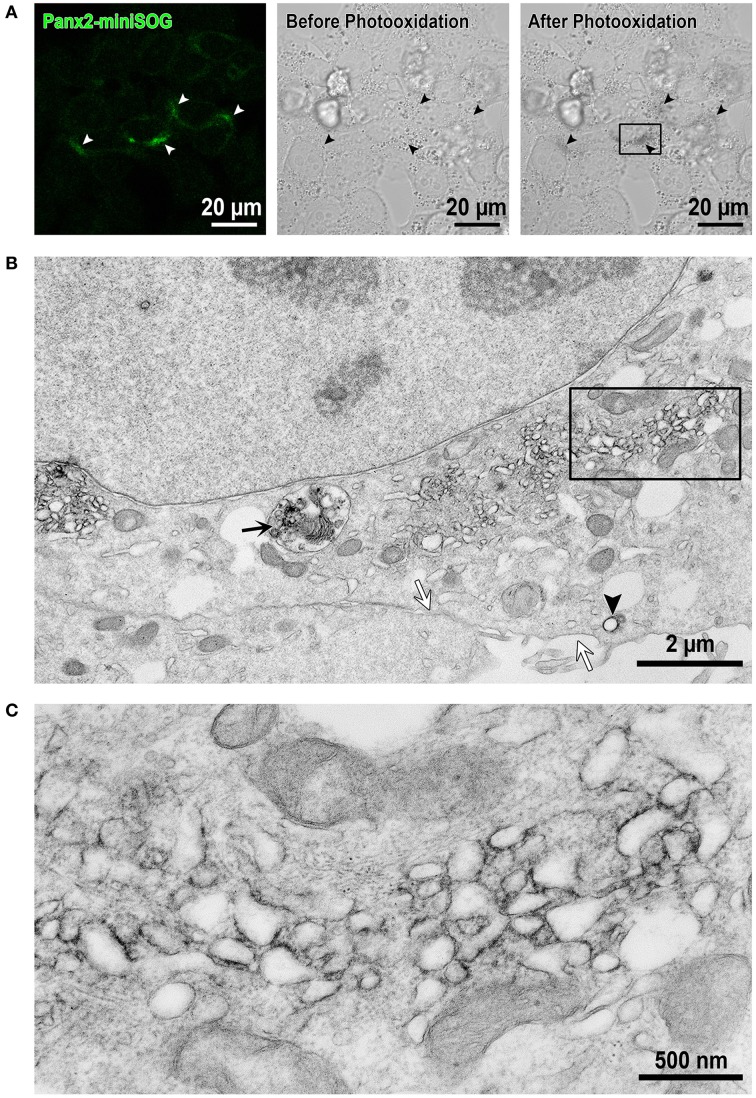
**EM imaging reveals intracellular localizations of Panx2-miniSOG in membrane bound structures. (A)** Confocal image from Panx2-miniSOG transiently expressed in HeLa cells (*left image*, white arrowheads point to miniSOG fluorescence). DIC images of the same area before (*middle image*) and after photooxidation (*right image*) with black arrowheads highlighting the same areas as in the fluorescence image. **(B)** Displayed is a low magnification EM thin section (corresponding to the boxed area in the right hand post-photooxidation DIC image in **A**). The black arrow points to a Panx2-miniSOG containing lysosome while the black arrowhead indicates a stained Panx2-miniSOG vesicle. Note that the plasma membrane is unstained (white arrows) and thus, contains no Panx2-miniSOG channels. **(C)** A higher four-fold magnification view of the boxed area in **(B)** revealed Panx2-miniSOG in intracellular tubulo-vesicular membranous compartments and minimal ER localizations.

In order to investigate the localization of Panx2 at high resolution, we took advantage of EM imaging in combination with protein specific staining to obtain increased spatial resolution of the labeled proteins within their cellular context. Specifically, we used a fusion protein composed of Panx2 and miniSOG for highlighting the specific distribution of Panx2. MiniSOG is a portion of the phototropin-2 protein from the plant *Arabidopsis thaliana* that has been genetically engineered for the specific purpose of using its diaminobenzidine (DAB) reactive properties to identify protein fusions by CLEM (Shu et al., 2011). Unlike immunolabeling techniques, this method has the advantage that strong fixatives can be used to optimally preserve cellular ultrastructure, a particularly important feature when analyzing membrane bound intracellular compartments. Panx2-miniSOG was transiently expressed in HeLa cells. The miniSOG tag is weakly fluorescent as shown in Figure 5A left image (highlighted by the white arrowheads), and following strong illumination with blue light it generates enough singlet oxygen to catalyze the deposition of osmophilic DAB polymers onto the tagged Panx2. Figure 5A middle and right images shows before and after photooxidation transmitted light micrographs. The black arrowheads identify the same cells as in Figure 5A left image (white arrowheads) that expressed the fluorescent Panx2-miniSOG. These transmitted light micrographs are useful for monitoring the photooxidation process, correlating the appearance of the optically visible DAB reaction product with the detected miniSOG intrinsic fluorescence, and for tracking the same areas in EM images. The osmium/DAB complex then serves as an electron dense label for EM. In the electron micrograph presented in Figure 5B, Panx2-miniSOG appears dark within intracellular membrane bound structures. This area is a higher magnification view of the boxed portion of cytoplasm between the nucleus and plasma membrane in Figure 5A right image. Within this region, labeled structures include elongated membranous portions, individual vesicles (black arrowhead) and a degradation compartment (black arrow) is readily apparent. In contrast, staining is not observed at the level of the plasma membrane (white arrows). A four-fold enlargement of the boxed area in Figure 5B, displayed in Figure 5C, highlights the cytoplasmic protrusions from the Panx2 oligomer seen in these intracellular membrane cross-sections.

#### Panx2 channels colocalize in early or recycling endosomes

**Figure 6:**
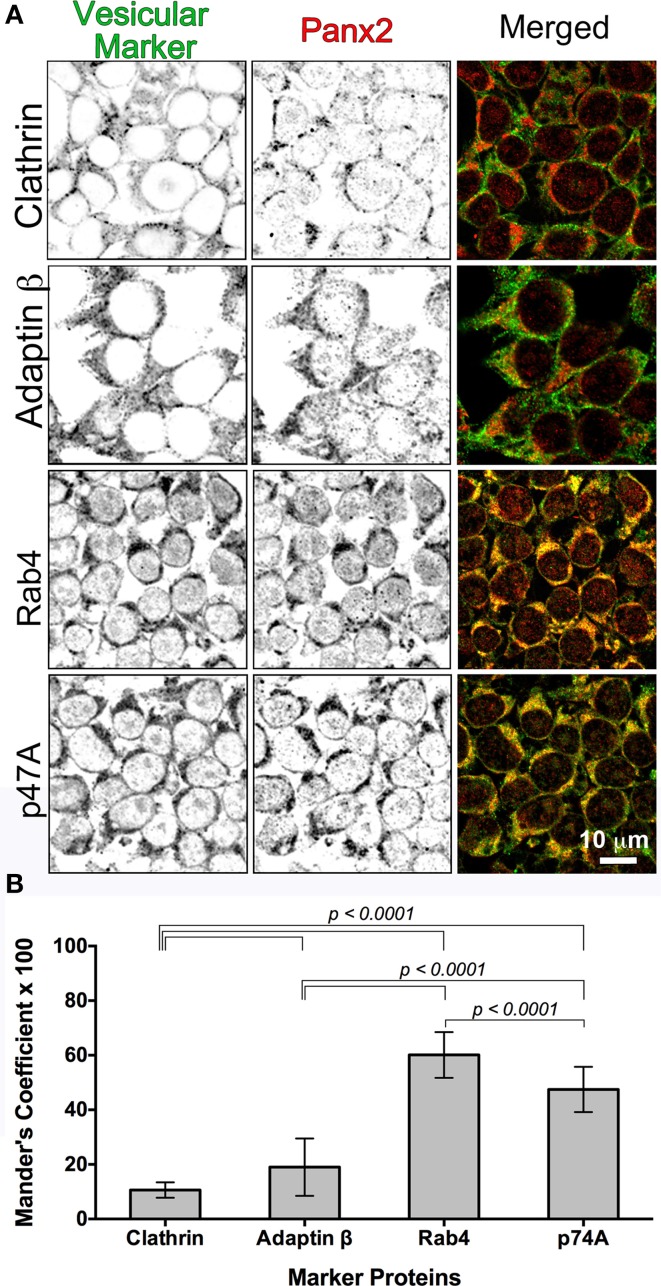
**Colocalization of Panx2 with cellular markers indicates early endosomal sorting**. In **(A)**, untagged WT Panx2 (red) and four organellar markers (green) were co-immunolabeled with the polyclonal anti-C terminal Panx2 antibody and a monoclonal antibody specific to each organellar proteins. The four protein markers, Clathrin, Adaptin β, Rab4 and p47A label clathrin-coated vesicles, early/recycling endosomes, early endosomes, and degradation vesicles derived from the Golgi, respectively. Both the single plane confocal images **(A)** and the Percent Colocalization (Manders' Coefficient × 100) graph in **(B)** indicated that Panx2 overlaps with early endosomes. Sample sizes for Manders' Coefficient calculation: 107 (clathrin), 119 (rab4), 131 (adaptin β) and 144 (p47A) cells. The errors bars indicate standard deviations.

**Figure 7:**
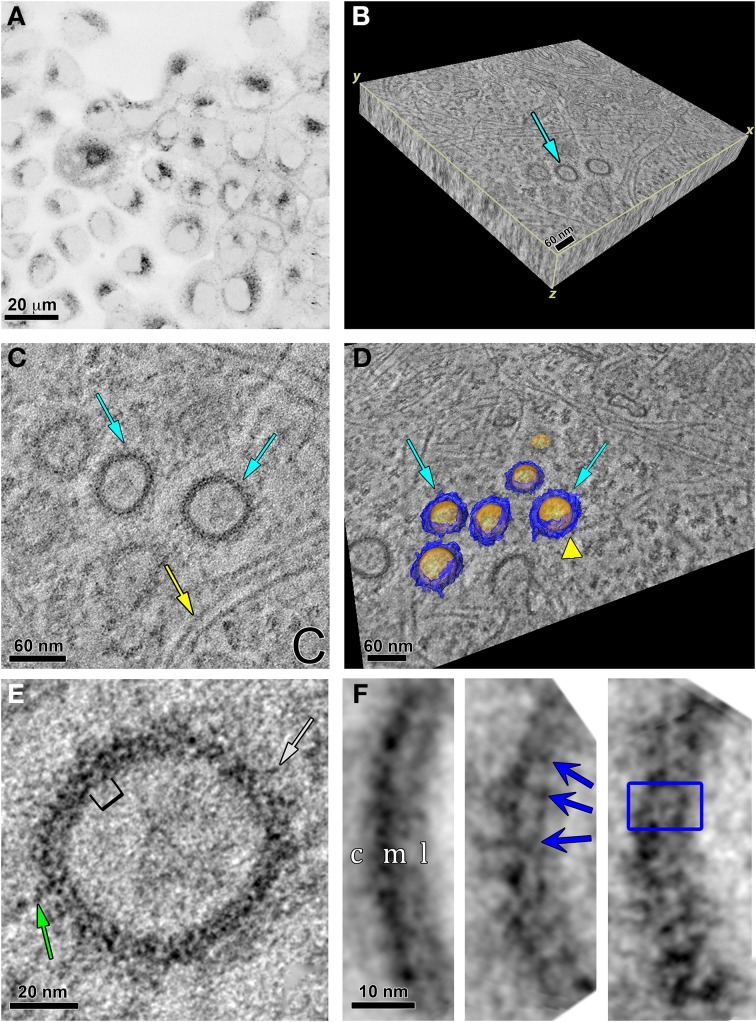
**Specific labeling of Panx2-4Cys and electron tomography highlight Panx2 distribution in the membrane of intracellular vesicles. (A)** MDCK cells stably expressing Panx2-4Cys were stained with ReAsH-EDT_2_, fixed and imaged with a confocal microscope before photooxidation. The image is displayed with an inverted color table (black is the highest fluorescence signal, white is no signal). **(B)** 3D representation of the EM tomographic volume as a 3D slab. Cyan arrow points to vesicles containing Panx2-4Cys oligomers. **(C)** Single X-Y slice from the tomogram showing Panx2-4Cys labeling in vesicle cross-sections (cyan arrows). Note the actin filaments in close proximity to the Panx2-4Cys-labeled vesicles (yellow arrow). **(D)** Automated segmentation of Panx2 vesicles (cyan arrows): yellow represents the lipid bilayer of the vesicle, while blue highlights the large domains of the Panx2 oligomers protruding from the vesicle membrane into the cytoplasm. The yellow colored arrowhead indicates an actin filament in close apposition to a vesicle. Based on comparisons with connexin channels, these protrusions most likely are from the large cytoplasmic domains of Panx2-4Cys. **(E)** Single slice of the tomogram showing a subarea of a Panx2-4Cys containing vesicle. Here stain-excluding areas (protein and lipid) are white and stain is black. The green arrows point to stained fine protrusions from the cytoplasmic surface of the vesicles. The bracket indicates an area bounded by stain that is ~8 nm. By analogy with connexin hemichannels, this distance should correspond to approximately the diameter through the membrane bound portion of Panx2 channels. **(F)** Three representative class averages obtained by single particle analysis of boxed cross-sectional areas. In the left hand average, c, cytosolic side; m, membrane; l, lumen of vesicle. Note the thicker cytosolic staining due to the deposition of DAB/osmium to the side containing the 4Cys/ReAsH tag. The blue arrows in the middle average and box in right hand average highlight substructure defined by stain from the Panx2 pore within the membrane bound portion (right hand average: inside box dimensions = ~8 × 10 nm).

D'Hondt et al. (2011) asserted in a review of intracellular functions of pannexin channels that pannexons may serve an endoplasmic reticulum (ER) Ca^2+^ release function. Their hypothesis was based mainly on a previous report that Panx1-EGFP stably overexpressed in human prostate cancer epithelial LNCaP cells had a fluorescent ER localization pattern overlapping with BODIPY-Brefeldin A fluorescence (Vanden Abeele et al., 2006) that correlated with Ca^2+^ leakage from the ER with tagged and untagged Panx1 ER expression. While we originally found that double immunolabeling of Panx2 and the ER marker protein calnexin in Panx2 transfected tissue culture cells revealed diffuse intracellular staining and significant overlap with calnexin (data not shown), suggesting that Panx2 localized in the ER, the CLEM data shown in Figures 5, **7** revealed instead that Panx2 is localized in the membrane of intracellular vesicles suggestive of endosomal systems and not in the ER. Endosomes represent sorting compartments within cells that serve to move proteins from/to the plasma membrane, lysosomes, and/or the Golgi apparatus. They are characterized as early, late and recycling endosomes. Because we saw no evidence for anterograde trafficking of Panx2 to the plasma membrane and a previous publication reported Panx2 being localized to the endolysomal system (Wicki-Stordeur et al., 2013), we performed immuno-colocalizations between Panx2 and endosomal constituent proteins used as markers for various endosomal compartments (Figure 6A). Four endosomal proteins were chosen for our colocalization experiments: Clathrin (endocytic vesicles), Adaptin β (protein sorting from the TGN and endosomes as well as clathrin-mediated endocytosis) (Boehm and Bonifacino, 2002), Rab4 (early and recycling endosomes) and p47A (vesicles derived from the Golgi). p47A is also known as AP3M1, a rat homolog of clathrin-associated adaptor proteins that interacts with the tyrosine-based sorting signal in the trans-Golgi network (Dell'Angelica et al., 1997). The monomeric GTPase Rab4 is associated with early endosomes, regulates recycling vesicle formation and provides for vesicle sorting, before it reaches lysosomes for degradation (Mellman, 1996; Mohrmann et al., 2002).

We expressed Panx2-WT (untagged) in HEK 293T cells and as expected, all four vesicular markers showed diffuse cytoplasmic fluorescence distributions. Overlap of Panx2-WT and vesicular markers is indicated by the yellow color in the merged Figure 6A single plane confocal images. For Clathrin and Adaptin β, this percent overlap (Manders' Coefficient × 100) was low at ~10–19% (*n* = 107 and *n* = 131 cells, respectively), whereas the percent overlap between Panx2 and Rab4 signals was statistically significantly higher at ~60% (*n* = 119) and ~48% for p47A and Panx2 (*n* = 144 cells) (Figure 6B). Thus, the high percentage overlap indicated that Panx2 is most likely localized to early or recycling endosomes.

#### 3D visualization by electron tomography demonstrated that intracellular vesicles contain Panx2 channels

In order to obtain a higher resolution view of Panx2 channels within intracellular vesicles, we used a fusion protein of Panx2 with the current generation of tetracysteine tag (Martin et al., 2005). Tetracysteine tags (4Cys) are small (~2 kDa), but require an exogenous biarsenical ligand for visualization. In tissue culture cells, diffusion of this ligand and binding to 4Cys is efficient and the red fluorescent biarsenical ReAsH ligand gives good photooxidation-driven DAB reaction product. Since DAB deposition is a localized reaction, we have found that DAB/osmium labeling better defines macromolecular complexes the closer the reaction occurs to the protein. We stably expressed Panx2-4Cys in MDCK cells, a cell line we have previously found to be particularly amenable to CLEM imaging and suffer less from over-expression of connexin superfamily proteins than HEK 293T or HeLa cells (Boassa et al., 2007, 2010).

As displayed in the fluorescence image in Figure 7A, the ReAsH labeling was localized intracellularly. This area was photooxidized and the sample dishes processed for EM. Using triple tilt electron tomography of 0.5 μm thick sections from these photooxidized areas, we obtained a tomographic volume of a portion of the cell containing vesicles (Supplemental Movie). The volumetric slab is shown in Figure 7B. Three vesicles are shown in cross section in a single slice from the tomogram (Figure 7C). Two of these are highlighted by cyan arrows while many actin filaments are found in close proximity (yellow arrow) or apposition in these vesicles (yellow arrowhead in Figure 7D). This is not surprising as Ohashi et al., showed that actin filaments regulated by cortactin was required for segregation of early endosomes in the perinuclear area, inducing movement of each endosome toward the cell center and preventing fusion events (Ohashi et al., 2011). Automated segmentation based on intensity thresholding using Amira was used to outline the sphere-like vesicles (colored in gold) and the proteinaceous coat (colored in blue) that surrounded the membrane boundary of these vesicles (Figure 7D).

Significant structural details were seen in the vesicle cross-section in this and other similar tomograms. A higher magnification view of one of the vesicles is displayed in Figure 7E and striations perpendicular to the membrane bilayer were apparent. We measured the diameters of these vesicles from the cross-sections in the tomogram. The vesicle population was variable with average ~88 ± 12.9 nm standard deviation (range 54.8–109.2 nm, *n* = 46 vesicles). The vesicles were not always spherical as we found the average ellipticity was 1.12 ± 0.15 in these cross-sectional views. Thus, sub-tomogram averaging would not have yielded a 3D consistent vesicle structure. Instead, we selected 128 × 128 pixel areas of vesicle membrane cross-sections from 2D slices in the full-resolution tomogram and used class averaging (Tang et al., 2007) to enhance any repeating substructure. In Figure 7F, we present three class averages out of 16. The left hand average has less defined repeating membranous substructure, but shows the thicker layer of staining on the cytosolic side of the vesicle, most likely due to the extensive Panx2 cytoplasmic domains and tag position. As defined by the staining, the membrane thickness was ~3.5–4 nm. The middle and right hand averages showed more variations in the membrane bound region and at the cytosolic surface. The dark striations we interpret as stain within the channel pore. The blue box represents an area of about 8.0 × 10.0 nm, dimensions that are reasonable for a Panx2 channel based on our own publication (Ambrosi et al., 2010) and the measured dimensions of connexin hemichannels from crystallographic analyses (Maeda et al., 2009; Oshima et al., 2011).

## Discussion

In contrast to the wealth of literature on the functionality of Panx1 and Panx3 as putative ATP channels, the functional role of Panx2 channels remains unresolved. Penuela et al. (2014) postulated that Panx2 may have ATP release functions similar to Panx1 because of a putative C-terminal caspase3 and caspase7 cleavage site analogous to a Panx1 caspase site that causes ATP release upon cleavage (Chekeni et al., 2010; Dourado et al., 2014). Nonetheless, it is clear from the data presented in this study and previously published studies by other laboratories (Lai et al., 2009; D'Hondt et al., 2011; Wicki-Stordeur et al., 2013; Le Vasseur et al., 2014) that Panx2 trafficking behavior and endolysosomal localizations are markedly different from Panx1 and Panx3 (Boassa et al., 2007; Penuela et al., 2007).

### Subcellular analysis of Panx2 and Panx1 in model cell systems and brain tissue showed similar segregation patterns

We used immunofluorescence imaging to confirm that Panx2 and Panx1 trafficked to intracellular compartments and the plasma membrane, respectively. The highly segregated populations of Panx1 and Panx2 in our light micrographs indicated that heteromeric combinations usually do not form. Our results are contrary to the co-immunoprecipitation and light microscopy imaging experiments of Penuela et al. (2009) and are confirmatory of our earlier hypothesis that Panx1/Panx2 channels are unstable *in vitro* (Ambrosi et al., 2010). Thus, it is unlikely that heteromeric channels would be found in native, unperturbed organ systems.

Because conventional fluorescence confocal microscopy does not provide sufficient resolution to examine details of subcellular compartments below the diffraction limit and provides information just based on signal from the probe, we performed EM on tissue culture cells expressing Panx2 C-terminally tagged with either miniSOG or 4Cys and reacted such that Panx2 could be easily identified within the cytoplasm at nanometer resolution (Figures 5, 7). We found strong staining in vesicle associated compartments, consistent with endolysosome distributions previously identified by Wicki-Stordeur et al. (2013). Sites for both endocytotic recognition and endolysosomal targeting sequences have also been identified in the Panx2 sequence using consensus sequence analysis programs (Boyce et al., 2014). Two endolysomal targeting sequences were identified in the intracellular loop (residue 153–158) and a distal part of the C-terminus (amino acids 647–652) while an area of the C-terminus sequence contained an endocytotic recognition sequence at amino acids 376–379. Rarely do we see Panx2 EM localizations in the ER as suggested by co-localization studies that solely used light microscopy for imaging (D'Hondt et al., 2011).

The endosomal colocalization experiments we presented indicated that expression patterns were consistent with early endosomal vesicle populations. The fact that we observed vesicle distributions in different cell types as well as in transient transfections and stable Panx2 expressing cell lines confirmed that this mode of trafficking is not an expression artifact. While early endosomes have typically been associated with endocytotic events (Jovic et al., 2010), early and recycling endosomes are now thought to act as entry portals, sorting stations, and signaling platforms. This mechanism provides an alternative route in biosynthetic pathways from traditional anterograde vesicular trafficking (De Matteis and Luini, 2008; Gould and Lippincott-Schwartz, 2009). The vesicular membrane cross-sections in our tomographic analysis contained Panx2 channel structures within the vesicle membrane and with distinct extensions into the cytosol (Figure 7) that are consistent with what is known about Panx2 protein topology. These images are very different from CLEM images of Panx1 channels we previously published in Boassa et al. (2007). Interestingly, endosomal cargo recycling typically involves the actin cytoskeleton (Gautreau et al., 2014), consistent with our observations of close associations of actin filaments in the tomograms.

In the present study, we observed strong non-overlapping Panx1 and Panx2 expression in both astrocytes and neurons in the adult mouse brain (Figure 4). While the Panx2 monomer has a similar molecular folding, it is much larger (~74 kDa; 674 amino acids) than Panx1 (~48 kDa; 426 amino acids) or Panx3 (~45 kDa; 392 amino acids). Its sequence is more distantly related to Panx1 and Panx3 than those proteins are to each other (Baranova et al., 2004). The mRNA for Panx2 is found in significant quantities in the CNS, both in astrocytes and neurons (Baranova et al., 2004; Bargiotas et al., 2011). Panx1 and Panx2 have a widespread and similar mRNA distribution, but Panx1 and Panx2 mRNA levels are inversely regulated during the development of the rat brain with significantly increase in Panx2 expression during post-natal development (Vogt et al., 2005). Small amounts of mRNA expression have been demonstrated in other tissues such as thyroid, prostate, liver and heart (Bruzzone et al., 2003). At the protein level, in cochlea immuno-localizations, Panx2 labeling was restricted to the basal cells in the stria vascularis and was also detected in the spiral ganglion neurons, but no overlapping labeling for Panx1 and Panx2 was observed (Wang et al., 2009).

Several studies have attempted to define a relationship between Panx2 within brain tissue and conditions that would up-regulate it. A gene array analysis showed an overall reduction of Panx2 in gliomas and a direct correlation was observed between Panx2 expression and post-diagnosis survival in patients (Litvin et al., 2006). Human Panx2 DNA is located in the same chromosomal regions that are implicated in human gliomas (Ino et al., 1999; Oskam et al., 2000; Hu et al., 2004) and exogenous Panx2 transfected into rat C6 glioma cells displayed a flattened morphology and increased cell–cell contacts and significantly reduced *in vitro* oncogenicity parameters (Lai et al., 2009). Zappala et al. (2007) showed that Panx2 was exclusively expressed by neurons of normoxic astrocytes–neurons primary co-cultures. Conversely, after 20 min of hypoxia, Panx2 was strongly expressed also in astrocytes. The *de novo* expression of Panx2 in astroglial cells after ischemia was proposed to play a role in the neuroprotection after ischemia (Zappala et al., 2007). A recent study showed that hippocampal seizure activity induced by a seizuregenic protocol using Co^2+^ up-regulated Panx2 mRNA and protein expression (Mylvaganam et al., 2010).

### Post-translational modifications of Panx2

Several post-translational modifications are possible based solely on analysis of the Panx2 sequence (Penuela et al., 2014). During brain development Panx2 has been described to be dynamically expressed over the course of post-natal hippocampal neurogenesis *in vivo* and *in vitro* with changes observed between intracellular and plasma membrane localizations associated with S-palmitoylation. Specifically, Panx2 is palmitoylated and localized intracellularly in neural progenitor cells and de-palmitoylated and at the plasma membrane in mature neurons (Swayne et al., 2010). These authors hypothesized that S-palmitoylation may regulate Panx2 intracellular sorting and protein-membrane interactions during neural development. Contrary to Panx1 and Panx3 where a complex glycosylated product (whose band on a western blot we originally named GLY2) (Boassa et al., 2007) was expressed at the plasma membrane, Panx2 was reported to exist in a high mannose form (GLY1) that exhibits only slight band shifts from the non-glycosylated form (GLY0) (Penuela et al., 2009). In this study, we have confirmed the existence of two forms for untagged Panx2 proteins. However, the tagged Panx2-HA exists only as a non-glycosylated core protein showing no change in the banding pattern after PNGase treatment; similarly dephosphorylation by alkaline phosphatase was ineffective indicating minimal or no post-translational modifications.

## Conclusions

Taken together our studies demonstrate that Panx2 is differently trafficked from Panx1. This endolysosomal trafficking mechanism implies that Panx2 has a different regulation mechanism than the other pannexins. While future studies may or may not establish ATP release as its functional role, significant Panx2 expression levels in the CNS and its concentration in the membrane of intracellular vesicles might suggest that latent Panx2 channels may require an external stimulus or insult to the cell that causes transport to the cell surface in fulfillment of its functional role.

### Conflict of interest statement

The authors declare that the research was conducted in the absence of any commercial or financial relationships that could be construed as a potential conflict of interest.

## Supplementary material

Supplemental Movie **Animation showing sequences of (1) one tilt series used for the triple tilt series tomography, (2) X-Y slices through the triple tilt tomogram and (3) automated segmentation of six vesicles (*gold = membrane; blue is proteinaceous material extending from the membrane into the cytoplasm*)**. The small black dots at the top and bottom of the tomogram and in the tilt series are gold beads deposited on the top and bottom of the section that are used for fiducial alignment, beam and section distortion corrections in the 3D reconstruction process (see Materials and Methods). Data, metadata, segmentation and the full resolution movie for this tomogram can be downloaded from the Cell Centered Database (CCDB MPID:48720 PID:2010) as noted in the Materials and Methods section.

## Abbreviations

Panx1: pannexin1
Panx2: pannexin2
CNS: central nervous system
EM: electron microscopy
CLEM: correlated lig and electron microscopy.
